# Survivin siRNA对人肺腺癌细胞SPCA1和SH77抑制作用的比较分析

**DOI:** 10.3779/j.issn.1009-3419.2011.01.04

**Published:** 2011-01-20

**Authors:** 全喜 刘, 超颖 董, 丽君 李, 捷 孙, 春云 李, 亮 李

**Affiliations:** 1 056002 邯郸，邯郸市第一医院胸外科 Department of Thoracic Surgery, the First Hospital of Handan City, Handan 056002, China; 2 056002 邯郸，邯郸市第一医院脑外二科 No.2 Department of Brain Surgery, the First Hospital of Handan City, Handan 056002, China; 3 050016 石家庄，河北 师范大学信息技术学院 College of Information and Technology, Hebei Normal University, Shijiazhuang 050016, China; 4 050017 石家庄，河北医科大学科技总公司 Company of Science and Technology, Hebei Medical University, Shijiazhuang 050017, China; 5 050016 石家庄，河北师范大学生命科学学院学院 College of Life science, Hebei Normal University, Shijiazhuang 050016, China

**Keywords:** 小干扰RNA, Survivin蛋白, 凋亡, 肺肿瘤, Small interfering RNA, Survivin protein, Apoptosis, Lung neoplasms

## Abstract

**背景与目的:**

Survivin是IAP家族的新成员，具有抑制凋亡和调节细胞增殖的双重作用，是迄今发现最强的凋亡抑制因子。本项研究的目的是通过将survivin的小干扰RNA（small interfering RNA, siRNA）表达载体分别转染至肺癌细胞SPCA1和SH77，分析survivin siRNA对不同肺癌细胞的增殖抑制作用。

**方法:**

构建靶向survivin的siRNA表达载体与pSi scrambled，经由脂质体法分别转染至肺癌细胞SPCA1和SH77。通过MTT法检测细胞增殖抑制，用流式细胞仪检测细胞凋亡率和细胞周期，利用半定量RT-PCR和Western blot印迹法分别检测细胞survivin mRNA及蛋白表达水平。

**结果:**

Survivin siRNA干预后，SPCA1和SH77细胞均出现凋亡，G_0_/G_1_期阻滞。实验组Survivin mRNA水平和蛋白表达水平比脂质体对照组明显减少。

**结论:**

*Survivin*基因特异性RNA干扰可以明显抑制肺癌SPCA1和SH77细胞的体外增殖并诱导明显的细胞凋亡。

## Abstract (en)

**Background and objective:**

Survivin, a member of the inhibitor of apoptosis (IAP) protein family, has been demonstrated as a potential new target for apoptosis-based therapy in cancer and lymphoma. The aim of this study is to investigate effects and mechanisms of survivin siRNA transfection on lung adenocarcinoma cell lines SPCA1 and SH77.

**Methods:**

A siRNA plasmid expression vector and pSi scrambled against survivin were constructed and transfected into SPCA1 and SH77 cells with Lipofectamine 2000. The proliferations of lung adenocarcinoma SPCA1 and SH77 cells were detected by MTT. The apoptotic rate and cell cycle were detected by flow cytometer. The activity of survivin mRNA and protein expression were analyzed with RT-PCR and Western blot.

**Results:**

Survivin siRNA reduced the proliferation of SPCA1 and SH77 cells. Cell cycle was inhibited in G_0_/G_1_. Expressions of survivin siRNA mRNA and protein were reduced in transfected cells compared with the control cells.

**Conclusion:**

siRNA targeted against survivin can effectively suppress SPCA1 and SH77 cells proliferation and significantly induce SPCA1 and SH77 cells apoptosis.

肺癌为常见的恶性肿瘤之一，数十年来肺癌的发病率和死亡率有明显的增高趋势^[1]^。Survivin是凋亡抑制蛋白（inhibitor of apoptosis family of protein, IAP）家族新成员^[2]^，可选择性地表达于恶性肿瘤组织中，而在除胸腺和生殖腺外的正常成人组织中不表达，这种特点使其成为目前恶性肿瘤诊断和治疗的新靶点。近年来，随着肿瘤分子生物学的发展以及对基因功能的深入研究，人们逐渐认识到基因治疗后对肿瘤细胞命运起主要决定作用的是基因型，而非制剂的基因毒性，肿瘤细胞相关基因的表达水平是造成患者对同一化疗药物敏感性不同的主要原因^[3]^。迄今为止，尚无科学研究比较分析不同肺癌细胞株小干扰RNA（small interfering RNA, siRNA）抑制survivin表达的效果。因此，本实验将survivin siRNA转染两种肺癌细胞SPCA1和SH77，检测其对肺癌细胞增殖抑制作用的不同。

## 1 材料与方法

### 1.1 实验材料

根据真核表达载体pSi-survivin和pSiscrambled的构建^[4]^设计survivin siRNA和scrambled对照cDNA序列，以pGCsi U6/Neo/GFP为载体，构建表达载体测序鉴定。Survivin siRNA靶基因的cDNA序列（编码基因368-386）为5′-GCAGTTGAAGAATAAC-3′。

人肺腺癌细胞SPCA1和SH77来源于美国模式培养物集存库（American Type Culture Collection, ATCC）。用含10%小牛血清RPMI-1640培养基，其内加入2 mmol/L的谷氨酰胺+0.05 mmol/L 2-巯基乙醇（2ME）+10%胎牛血清（胎牛血清）。保持细胞浓度为2×10^4^个/mL-9×10^4^个/mL，在37 ℃、5%CO_2_饱和湿度的条件下培养。用甘油作为其冷冻保护剂。2.5 g/L胰酶消化传代。实验时取对数生长期细胞。首先将细胞在10%FCS中培养2 d。然后每2天通过离心、洗涤6次且以传代的方式收集细胞。

利用Qiagen去内毒素质粒提取试剂盒，大量提取去内毒素的重组质粒pSi-scrambled和pSi-survivin。接种SPCA1和SH77细胞于100 mL培养瓶中，细胞数约1×10^6^个，用10%新生牛血清但不含抗生素的RPMI-1640培养液，在37 ℃培养至细胞占瓶底约80%-90%时，按Invitrogen公司的Lipofectamine 2000使用说明书进行转染，质粒和转染试剂的比例为2:1。转染分组情况如下：①脂质体对照组；②pSi-scrambled对照组；③pSi-survivin SPCA1细胞实验组；④pSi-survivin SH77细胞实验组。

### 1.2 MTT法^[5]^检测细胞增殖抑制

将SPCA1和SH77细胞种在96孔板中，100 mL培养基中1×10^4^个细胞。将细胞分为3组，分别转染空脂质体、pSi-scrambled和pSi-survivin质粒。细胞在37 ℃环境下培养48 h。每个孔加入50 mL的MTT溶液。培养4 h后，样品溶于二甲基亚砜（dimethyl sulfoxide, DMSO）中，并用酶标仪检测样品吸光度，测定波长492 nm，参考波长690 nm。光密度（optical density, OD）值表示试验组和对照组的光密度。重复实验3次。采用线性分析确定IC_50_值。

### 1.3 流式细胞仪检测细胞凋亡率和细胞周期

SPCA1和SH77细胞培养为5×10^5^个细胞/mL的细胞浓度，3个重复。孵育48 h后，转染空脂质体、pSi-scrambled和pSisurvivin质粒处理的细胞进行平行对照。细胞用70%乙醇收集并悬浮（4 ℃、1 h）。用胰蛋白酶进行收集并用PBS液冲洗3次。将悬浮颗粒置于250 mL PBS和100 mL Annexin V/PI溶液中。在避光室温情况下碘化染色30 min，细胞悬浮液通过流式细胞仪（BD公司，美国）进行检测。测定细胞周期，观察G_0_/G_1_期、G_2_/M期、S期各期细胞所占的百分比，观察是否存在凋亡峰。通过WinMDI 2.5版本软件（TSRI流式细胞术）来分析。

### 1.4 半定量RT-PCR检测survivin mRNA水平

转染72 h后，利用Trizol试剂（Inv itrogen公司）提取细胞总RNA，RT-PCR扩增目的基因*survivin*和内参基因*β-actin*。反应体系中加入两对引物，第一对是*survivin*基因的引物，即5′-GAATTCATGGGTGCCCCGACGTTGCC-3′和5′-AGATCTTTCTTCTTATTGTTGGTTTCC-3′，扩增片段长度为475 bp。第二对是*β-actin*基因的引物，即5′ -GTGGGGCGCCCCAGGCACCA-3′和5′-CTCCTTAATGTC ACGCACGATTTC-3′，扩增片段长度630 bp。PCR反应条件：94 ℃、30 s，59 ℃、30 s，72 ℃、60 s，30个循环后，72 ℃延伸10 min。PCR产物经1.5%琼脂糖凝胶电泳分离，紫外灯下拍照。

### 1.5 Western blot检测survivin蛋白含量

转染72 h后，通过缓冲液裂解制备SPCA1和SH77全细胞裂解液，缓冲液中含有1%Nonidet P-40、乙磺酸50 mmol/L（pH7.5）、氯化钠100 mmol/L、EDTA 2 mmol/L、焦磷酸缓冲液1 mmol/L、原钒酸钠10 mmol/L、苯甲基磺酰氟1 mmol/L以及氟化钠100 mmol/L。相同浓度的裂解液与十二烷基硫酸钠反应-10%聚丙烯酰胺凝胶电泳，并用转移缓冲液（Tris 25 mmol/L，甘氨酸192 mmol/L，甲醇10% [v/v]）将其转移到PVDF膜上。PVDF膜用Tris缓冲液（TBS: Tris10 mmol/L[pH7.4]，氯化钠150 mmol/L）冲洗，并用TBS-5%牛血清白蛋白（bovine serum albumin, BSA）在室温下过夜，含有抗体的TBS-5%BSA孵育。ECL化学发光试剂盒（Kirkegaard & Perry Labo-ratories）显色进行检测survivin的表达。

### 1.6 统计分析

采用SPSS 10.0统计软件进行分析。3次独立实验的数据采用Mean±SD表示，实验组与对照组的组间差异比较采用*t*检验分析，*P* < 0.05为有统计学差异。

## 2 结果

### 2.1 siRNA转染后抑制SPCA1和SH77细胞增殖

利用MTT法检测各组转染细胞的存活状态，以脂质体组的生存率作为100%，则pSi-scrambled对照组、pSi-survivin SPCA1实验组和pSi-survivin SH77实验组的生存率分别为95.2%、33.8%和36.4%，pSi-survivin SPCA1实验组和pSi-survivin SH77实验组出现了较大程度地生长抑制现象，与对照组相比较，均具有统计学差异。但是实验组组间差异不明显（图 1）。

**1:**
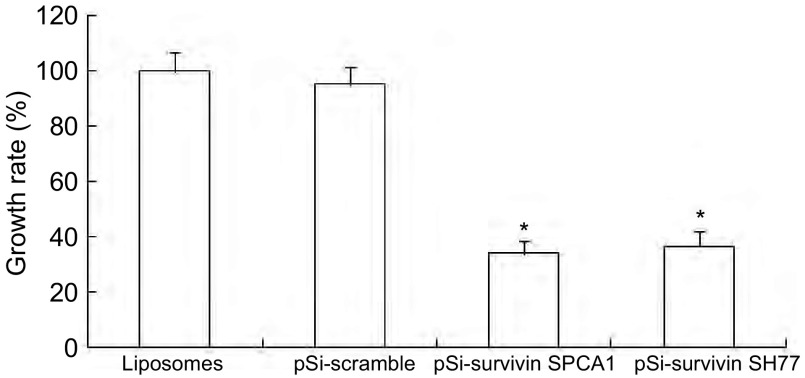
Survivin siRNA转染后抑制SPCA1和SH77细胞增殖。^*^：与对照组相比，*P* < 0.05。 The inhibition of SPCA1 and SH77 cells proliferation by survivin siRNA. ^*^P < 0.05, *vs* control group.

### 2.2 siRNA转染后诱导细胞凋亡并引起G_0_/G_1_细胞周期阻滞

pSi-survivin SPCA1实验组和pSi-survivin SH77实验组与脂质体对照组相比，出现了明显的细胞凋亡。与阴性对照组相比，pSi-survivin SPCA1实验组和pSi-survivin SH77实验组细胞的G_0_/G_1_期比例明显增加，S期细胞减少，G_2_期细胞明显减少，细胞阻滞在G_0_/G_1_期（表 1）。

**1:** Survivin siRNA诱导细胞凋亡和SPCA1和SH77细胞周期阻滞 The cell cycle distribution of SPCA1 and SH77 cells apoptosis and cell cycle inhibition by the survivin siRNA

Group	Ratio of apoptosis (%)	Cell cycle distribution (%)		
		G_0_/G_1_	S	G_2_/m
Liposomes	2.2±0.1	54.3±1.2	32.9±0.5	10.7±1.4
pSi-scramble	3.7±0.8	58.2±2.1	26.8±3.9	14.8±0.6
pSi-survivin SPCA1	38.8±3.6	73.6±3.7^*^	20.1±0.4^*^	6.3±1.5^*^
pSi-survivin SH77	33.1±2.0	74.1±2.7^*^	19.7±1.8^*^	6.1±1.3^*^
^*^P < 0.05, *vs* control group.				

### 2.3 siRNA抑制转染细胞survivin mRNA水平

转染后72 h提取细胞总RNA，利用半定量RT-PCR方法检测survivin基因转录水平（图 2A）。运用GIS凝胶分析软件光密度扫描分析，以脂质体对照组survivin产物与内参β-actin产物的电泳亮度比值定为1，则其它各组的比值分别为：pSi-scrambled对照组为0.57，pSi-survivin SPCA1实验组和pSi-survivin SH77实验组分别为0.76和0.33（图 2B）。经统计分析与脂质对照组相比，其它三组均明显降低。结果表明重组质粒pSi-survivin在mRNA水平上抑制了*survivin*基因转录。

**2:**
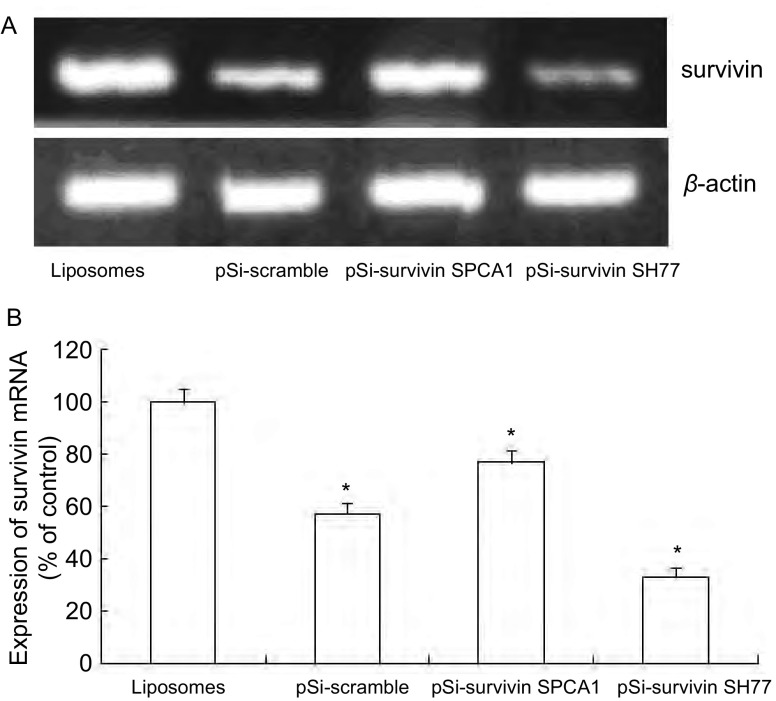
Survivin siRNA抑制SPCA1和SH77细胞survivin mRNA表达。A：RTPCR检测结果；B：柱状分析图表明survivin siRNA有效地抑制了两种细胞中*survivin*的基因表达。^*^：与脂质对照组相比，*P* < 0.05。 Expression of survivin mRNA in SPCA1and SH77 cell inhibited by survivin siRNA. A: Result of RT-PCR test. B: The analysis of survivin mRNA in SPCA1 and SH77. It shows that survivin siRNA inhibited survivin mRNA expression effectively. ^*^P < 0.05, *vs* Liposomes control group.

### 2.4 siRNA抑制细胞中survivin蛋白表达

与两对照组相比，pSi-survivin SPCA1实验组和pSi-survivin SH77实验组的survivin蛋白表达受到了明显抑制（图 3）。结果证实survivin siRNA可在蛋白水平抑制survivin表达。

**3:**
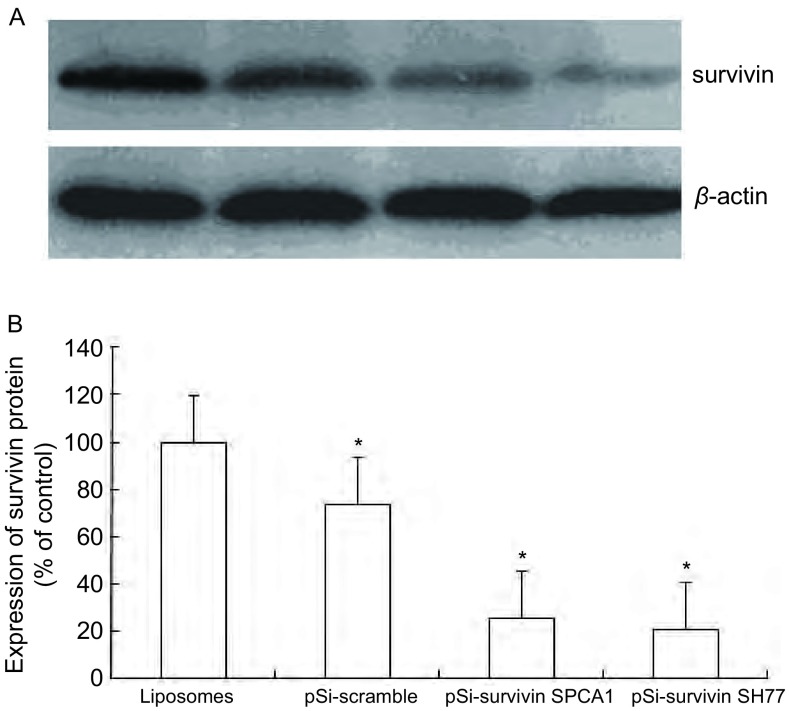
Survivin siRNA抑制SPCA1和SH77细胞survivin蛋白表达。A：Western blot检测结果；B：柱状分析图表明survivin siRNA有效地抑制了两种细胞中survivin的蛋白表达。^*^：与对照组相比，*P* < 0.05。 Expression of survivin protein in SPCA1 and SH77 cell inhibited by survivin siRNA A. Result of Western blot test; B: The analysis of survivin protein expression in SPCA1 and SH77. It shows that survivin siRNA inhibited survivin protein expression effectively. ^*^P < 0.05, *vs* control group.

## 3 讨论

中国是一个高发性肺癌的危险区。对原发性肺癌治疗仍然困难，并且依赖于基础医学研究。最近的研究^[6-8]^结果表明，细胞凋亡与肿瘤的发生、进展和转移密切相关。Survivin作为一种新发现的IAP，具有抗凋亡和调节细胞周期的双重功能，在人类众多恶性肿瘤中广泛表达，而在正常组织中不表达或低表达。目前，survivin已经成为非小细胞肺癌（non-small cell lung cancer, NSCLC）最显著的独立预后影响因子之一^[9-12]^。Survivin的选择性分布特点使其成为肿瘤基因治疗的理想靶点，针对*survivin*的基因治疗具有良好的靶向性、特异性和安全性，可以促进肿瘤细胞凋亡并抑制其增殖，但对正常组织几乎没有不良影响。

令人感兴趣的是，不同患者对相同化疗方案的治疗效果和反应有差别，特别是不同病理类型的肺癌患者对化疗药物的敏感性差异较大^[13-16]^。为避免治疗的盲目性，有必要探索不同细胞对化疗敏感或耐药的更深层的原因，以期为肿瘤的个体化疗提供依据。近年来，随着肿瘤分子生物学研究的发展，人们逐步认识到基因表达水平不但决定肿瘤发生、发展和预后，而且与化疗敏感性存在某种相关性。所以，本研究选择了2种类型的人肺癌细胞株作为对象，用survivin siRNA分别转染，检测其对肺癌细胞的细胞周期、mRNA和蛋白表达水平的不同，探讨同一种构建的*survivin*基因siRNA质粒对不同的细胞株是否有不同的作用效果。

本研究中我们构建了靶向*survivin*基因的siRNA表达质粒并转染肺癌细胞SPCA1和SH77，通过半定量RTPCR及Western blot检测结果表明，所构建的靶向survivin的siRNA表达载体具有高度的特异性，可有效地抑制SPCA1和SH77细胞中survivin mRNA及蛋白的表达；抑制SPCA1和SH77细胞*survivin*基因的表达可以显著抑制细胞的增殖并诱导明显的细胞凋亡。本研究与于振香等^[17]^的研究发现一致，即survivin的siRNA表达载体可抑制肺癌细胞A549的增殖，细胞受阻于G_0_/G_1_期，可抑制survivin mRNA及蛋白表达，并可诱导细胞凋亡。

可见，survivin siRNA可抑制多种细胞株的增殖，并可诱导细胞凋亡。有望进一步扩充细胞株，深入探讨其功能。
